# Chondroprotective Effects of a Standardized Extract (KBH-JP-040) from *Kalopanax pictus*, *Hericium erinaceus*, and *Astragalus membranaceus* in Experimentally Induced In Vitro and In Vivo Osteoarthritis Models

**DOI:** 10.3390/nu10030356

**Published:** 2018-03-15

**Authors:** Md. Mahbubur Rahman, Hyun-Kyu Kim, Seong-Eun Kim, Myung-Jin Kim, Do-Hyung Kim, Hak Sung Lee

**Affiliations:** 1 KNOTUS Co., Ltd. Research and Development Center, 189 Donggureung-Ro, Guri-Si 11910, Gyeonggi-Do, Korea; mahbubs84@gmail.com or mahbub@knotus.co.kr (M.M.R.); third_onezest@knotus.co.kr (M.-J.K.); lab@knotus.co.kr (D.-H.K.); 2 Kolmar BNH Co., Ltd. 22-15, Sandan-gil, Jeonui-myeon, Sejong-si 30003, Korea; hkkim@kolmarbnh.co.kr (H.-K.K.); kse1316@kolmarbnh.co.kr (S.-E.K.)

**Keywords:** *Kalopanax pictus*, *Hericium erinaceus*, *Astragalus membranaceus*, chondrocytes, arthritis

## Abstract

The aim of this study was to investigate the chondroprotective effect of a standardized extract (KBH-JP-040) of the Korean traditional herbs *Kalopanax pictus* Castor-Aralia, *Hericium erinaceus* (Bull.) Persoon, and *Astragalus membranaceus* Schischkin on in vivo and in vitro osteoarthritis (OA) models. Cultured rat chondrocytes were pre-treated with KBH-JP-040 (50, 100 and 200 μg/mL) for 1 h, then recombinant human IL-1α (rhIL-1α) for 24 h. For the in vivo model, rabbits (*n* = 60) were equally divided into experimental groups: normal control (NC), a collagenase-induced OA group, and OA groups treated with KBH-JP-040 (75, 100, and 150 mg/kg body weight) and celecoxib (Cx, 100 mg/kg) orally for 28 days. Treatment with KBH-JP-040 significantly attenuated inflammatory cytokines and matrix metalloproteinases (MMPs), suppressed the expression of IκBα, NF-κB, and JNK/p38 mitogen-activated protein (MAP) kinase, and upregulated aggrecan and collagen type-II expression in rhIL-1α-stimulated chondrocytes. Furthermore, the serum and synovial levels of inflammatory cytokines of rabbits also decreased in the treatment groups when compared with the OA group. Improved magnetic resonance imaging and histopathological findings further confirmed the therapeutic efficacy of KBH-JP-040 against OA. In conclusion, these results indicate that KBH-JP-040 possesses chondroprotective effects, suppressing inflammation and MMPs, and downregulating IκBα, NF-κB, and JNK/p38 MAP kinase-signaling pathways. This might be a potential therapeutic candidate for OA treatment.

## 1. Introduction

Osteoarthritis (OA) is a chronic degenerative disease characterized by synovial inflammation, cartilage destruction, bone-margin erosion, and the sclerosis of subchondral bones. It is related to several pro-inflammatory mediators and a series of complex mechanisms. Increased levels of pro-inflammatory cytokines, interleukins (ILs), matrix metalloproteinases (MMPs), lysozyme, hyaluronic acid, cartilage glycoprotein, and substance-P have been found in the serum or synovial fluid of osteoarthritic patients and experimental animals [1,2,3]. OA is the leading cause of disability in the world [4]. The prevalence rate of OA in people aged over 65 is about 60% in men and 70% in women [5,6]. Moreover, the occurrence of OA is increasing daily [7], with an estimated incidence of up to 100,000 new cases per year [8]. This is due to population aging, lifestyle, eating habits, obesity, malnutrition, occupational injury, and trauma [1,4,7,9]. 

Many drugs like analgesics, opioids, non-steroidal anti-inflammatory drugs (NSAID), and COX-2 specific drugs, are used in the treatment of OA [10]. However, currently available drugs for OA treatment have several side effects, including increasing the risk of cardiovascular/gastrointestinal diseases and adverse effects on cartilage [2]. Celecoxib is a COX-2 selective NSAID, which is commonly used as an effective drug to alleviate osteoarthritis. However, prolonged use of celecoxib may be associated with concomitant increase in vascular risk, including myocardial infarction [11]. Recent studies on the complementary treatment of OA have focused on traditional herbal medicines [2,3,12]. *Kalopanax pictus* (KP) [13,14], *Hericium erinaceus* (HE) [15], and *Astragalus membranaceus* (AM) [10] are widely used in Korean traditional medicine safely and reliably for the treatment of multiple diseases, including arthritis and inflammatory diseases. The major constituents of KP have been identified are liriodendrin, kalopanaxsaponin (A, B, C, D, E, F, G, H, I, J, and K), syringin, erythrakine etc. [16,17,18]. More than 100 compounds, including flavonoids (formononetin), saponins, polysaccharides, and amino acids have been identified in AM, and the various biological activities of the compounds have been described [19,20]. Many active components of HE have been detected, including polysaccharides and secondary metabolites such as betuline, erinacines, hericerins, hericenones, resorcinols, ergosterol peroxide, cerevisterol, inoterpene A, astradoric acid C, oleanolic acid, ursolic acid, hemisceramide steroids, mono- and diterpenes, and volatile aromatic erythrakine compounds, and nutritional components [21,22]. In general, these three herbs are rich in pharmacologically active polysaccharides, saponins, polyphenols, flavonoids, and alkaloids, which are directly involved in their therapeutic efficacy.

Various herbal plants or their products have been used alone or in combination in traditional medicine to treat different types of diseases, with considerable success, along with fewer or no side effects [3,14]. Although these herbs or their active components have been in use for a long time, and their medicinal applications and general safety are well known, in many cases, their combination for use as therapeutic agents has not been evaluated. Therefore, the aim of this study was to examine the effectiveness of a combination of three herbal agents in experimental rat chondrocytes and rabbit osteoarthritis (OA) model, that may help to reduce inflammation, protect cartilage damage, relieve pain, and improve joint pathology by comparison with celecoxib.

## 2. Materials and Methods 

### 2.1. Preparation of Standardized Extracts (KBH-JP-040)

Three batches (No. DHP20160630-20160704) of standardized extract (KBH-JP-040) containing 7.50% liriodendrin (KP), 0.11% betulin, (HE), and 0.01% formononetin (AM) as a marker compound were manufactured and verified by Daeho Corporation Co., Ltd. (Hwaseong, Korea). Briefly, the fresh fruit body of HE, the bark of KP, and the root of AM were collected, shattered, and dried separately. The three herbs were extracted by heating at 95 °C twice (for 4 h and 2 h) using distilled water. The extract was then filtered and concentrated (Busung Tech, Ansung, Korea) to 15–20 degrees Brix at 65 °C. The concentrated extract was spray dried (Mehyun Engineering, Anyang, Korea) at an inlet temperature of 170 ± 10 °C and outlet temperature of 80 ± 5 °C. For HE, after the second extraction, Brix was measured, and an equal amount of dextrin was added before spray drying in the same condition. The extraction yields were approximately 19% KP, 43% HE, and 35% AM. Finally, the extract was mixed in the ratio of HE/KP/AM = 15:70:15, and this combined extract powder (KBH-JP-040) was used for the experiment. 

### 2.2. Quantitative Analysis of Liriodendrin, Betulin, and Formononetin by HPLC

The marker compounds’ (liriodendrin, botulin, and formononetin) contents in the KBH-JP-040 were analyzed according to methods previously established by KolmarBNH. Powdered extracts and standards (98% HPLC purity, Sigma, St. Louis, MO, USA) were completely dissolved in methanol, and the solutions were filtered through a membrane filter (0.45 μm, Millipore, Darmstadt, Germany). Concentrations were determined by a HPLC system (UltiMate 3000 HPLC, thermo Dionex, Germering, Germany) with a flow rate of 1.0 mL/min. HPLC conditions for the quantification of each compound were as follows: Cosmosil (Nacalai Tesque, Kyoto, Japan) RP18 column (4.6 mm x 250 mm, 5 μm), methanol (including 0.04% TFA) elution solvent, and 210, 250 nm detection wavelengths. The injection volume was 10 μL for both standard and samples. Peaks were identified by comparison with the retention time of the standards.

### 2.3. Isolation and Culture of Chondrocytes

The animal study was approved by the Institutional Animal Care and Use Committee at the KNOTUS Co., Ltd., Guri-si, Korea (Certificate No.: IACUC 16-KE-048). The 3-week old Sprague–Dawley male rats (*n* = 6) were sacrificed by decapitation after anesthesia in an induction chamber by isoflurane 3.5% for 2–4 min. Chondrocytes were collected from under aseptic conditions. The cartilage was washed five times with phosphate-buffered saline (PBS) containing 10% penicillin–streptomycin (Gibco, Paisley City, UK) within 10 min, and then cut into small pieces. The mixture was primarily digested by 0.25% trypsin-ethylenediaminetetraacetic acid (EDTA) for 30 min, and subsequently with 0.2% collagenase II in Dulbecco’s modified Eagle’s medium (DMEM) for 1.5 h of enzymatic digestion at 37 °C. The articular chondrocytes were cultured in minimum essential medium (MEM) containing 10% fetal bovine serum (FBS), 2 mL l-glutamine, 50 U/mL penicillin, and 50 μg/mL streptomycin, and seeded in a 75 cm^2^ culture flask, then incubated at 37 °C, CO_2_ 5%. At about 90% confluence, the chondrocytes were passaged for number three. The chondrocytes were detached by 0.25% (*w*/*v*) trypsin, 0.53 mM EDTA solution (3 mL) after centrifugation (125 *g* for 10 min), and the separated chondrocytes were then used in this study. The cell populations were confirmed to be 1 × 10^8^ per rat. RNA isolation was confirmed after chondrocyte markers of collagen type-II or the expression of the *SOX9* gene using real-time polymerase chain reaction (RT-PCR) (Table 1) had been undertaken, in order to identify the stabilized chondrocytes. This was done to ensure the reliability of the cells used in the experiment. All experimental samples analyses are performed in triplicate, and mean data from each treatment were collected and used for statistical analysis.

### 2.4. Cytotoxicity Measurement and Treatment Procedures

The cytotoxicity effect of KBH-JP-040 in chondrocytes was evaluated by MTT assay. Cellular toxicity was not observed up to 200 μg/mL concentration. In this study, 0–200 μg/mL of KBH-JP-040 was employed. The KBH-JP-040 product obtained was diluted in culture in different concentrations (0, 50, 100, and 200 μg/mL) to treat the test subject with the cultured chondrocytes. In general, cell density was 5 × 10^4^ cells seeded/well in 500 μL volume in a 48-well plate. After 1 h of processing, each of the test materials were subsequently co-cultured with or without recombinant human IL-1α (rhIL-1α) (5 ng/mL) for 24 h, in order to evaluate the indicator substance for osteoarthritis. The application of rhIL-1α in chondrocytes creates a meaningful micro-environment of OA for in vitro studies by inducing the inflammation and degradation of cartilage [23,24].

### 2.5. Evaluation of Protein Level by Enzyme-Linked Immunosorbent Assay (ELISA)

After treatment of the chondrocytes with KBH-JP-040 (0, 50, 100, and 200 μg/mL) for 1 h and following co-incubation for 24 h with rhIL-1α (5 ng/mL) and aggrecan (MyBioSource, San Diego, CA, USA), collagen type-II content was assessed using a sandwich enzyme-linked immunosorbent assay (ELISA) kit (Chondrex, Inc., Redmond, WA). Prostaglandin E2 (PGE2) (R&D Systems Inc., Minneapolis, MN, USA). The levels of MMP-1, MMP-9 (R&D Systems Inc.), MMP-13 (MyBioSource), IL-6, IL-10 (eBioscience, San Diego, CA, USA), and IL-12 (MyBioSource) protein in culture medium were assayed with an ELISA kit (R&D Systems Inc.) following the manufacturer’s instructions.

### 2.6. Measurement of Protein Expression by Real-Time Reverse Transcriptase Polymerase Chain Reaction (Real-Time RT-PCR)

To investigate the effect of KBH-JP-040 on the osteoarthritis-related cellular gene expression of MMPs and ILs in chondrocytes lysates, real-time RT-PCR was used. Total cellular RNA was isolated from chondrocytes using easy BLUE (iNtRON, INC., Daejeon, Korea), and used in real-time RT-PCR with a CFX96TM detection system (Bio-Rad Laboratories, Hercules, CA, USA). Following reverse transcription of total RNAs with high-capacity cDNA reverse transcription kits (Applied Biosystems, Foster City, CA, USA), the reaction mixture contained 2 μL of template cDNA, 10 μL of 2× SYBR Primix Ex Taq, and 200 nM primers in a final volume of 20 μL. The reactions were denatured at 95 °C for 30 s, and then subjected to 45 cycles at 95 °C for 5 s and at 60 °C for 20 s. When the reaction cycles were completed, the temperature was increased from 65 °C to 95 °C at a rate of 0.2 °C/15 s, and the fluorescence was measured every 5 s to construct a melting curve. A control sample that contained no template DNA was run with each assay, and all determinations were performed at least in duplicate to ensure reproducibility. The authenticity of the amplified product was determined by melting-curve analysis. All data were analyzed using the Bio-Rad CFX Manager, version 2.1 analysis software (Bio-Rad Laboratories). GADPH was used as an endogenous control. All analysis had been performed in triplicate according to the technical replicate. The target cDNA was amplified using sense primers and antisense primers (Table 1).

### 2.7. Reactive Oxygen Species (ROS) Measurement

For reactive oxygen species (ROS) determination, chondrocytes were dispensed into 96-well plates in concentrations of 5 × 10^4^ cells/well. After stabilization for some time, the cells were treated with KBH-JP-040, as before, for 1 h, followed by co-incubation with rhIL-1α (5 ng/mL) for 24 h. After incubation, the cells were washed with PBS and stained with dichlorodihydrofluorescein (DCFH) diacetate (DCFD, 10 mol/L PBS) for 30 min in the dark. Next, cells were washed with PBS twice, and extracted with 0.1% Tween-20 in PBS (PBST) for 10 min at 37 °C. DCFH fluorescence was recorded using a spectrophotometer at an excitation wavelength of 490 nm and an emission wavelength of 525 nm.

### 2.8. Western Blot Analysis

After the treatment described above, the chondrocytes were washed with ice-cold PBS and lysed on ice with lysis buffer. The protein samples were separated by 10% sodium dodecyl sulfate-polyacrylamide gel electrophoresis. The separated proteins were then transferred onto polyvinylidene difluoride membranes. The membranes were saturated and blocked with 5% skimmed milk at room temperature for 1 h, then incubated with a primary antibody of p-IκB-α, IκB-α, p-NFκB, NFκB, p-p38, p38, p-JNK, JNK, and β-actin at 4 °C overnight. After extensive washing with PBST (with Tween-20), the second antibody was added. The detection of specific antibody–antigen complexes was carried out using a chemiluminescence (ECL) reagent (Thermo) by utilizing an image analyzer (Alpha innotech), and confirmed the identity of the band. Bands were quantified using the ImageJ program. Density levels of the phosphorylated form p- (e.g., p-IκB-α, p-NFκB, p-p38, p-JNK) and the total form (e.g., IκB, NFκB, Tues % share in p38, JNK) were compared with the control. 

### 2.9. Animal Experimental Design

Sixty male New Zealand white rabbits, (2.5–3.0 kg, Dream Bio, Seongnam, Gyeonggi, Korea) were used. They were housed separately in stainless steel cages (W 500 × L 800 × H 500 mm) in an environmentally controlled room (temperature of 23 ± 2 °C, relative humidity of 55 ± 15%, in a 12 h light/dark cycle of 150~300 Lux, ventilation 10–20 times/h). Food and sterilized water were available ad libitum. Animals were divided equally into 6 groups: NC, vehicle-treated normal control group; OA, vehicle-treated collagenase-induced osteoarthritis group; Cx100, collagenase-induced OA rabbits treated with celecoxib 100 mg/kg; collagenase-induced OA rabbits treated with standardized extract, KBH-JP-040-treated groups (75, 100, and 150 mg/kg body weight). After induction of arthritis, at the seventh day following initial collagenase injection, the extracts were diluted according to the dose, and animals were administered 5 mL/kg orally every day by latex catheter (Sewoon 11, Sewoon Medical Ltd., Seoul, Korea). All animals were closely monitored, and there were no clinical symptoms observed during the entire experimental period. This animal study was also approved by the Institutional Animal Care and Use Committee at the KNOTUS Co., Guri-si, Ltd., Korea (Certificate No: IACUC 15-KE-130).

### 2.10. Induction of Arthritis

After the administration of anesthesia, Zoletil 50 (VIRBAC, Carros, France, 5 mg/kg), and xylazine (Rompun^®^, Bayer AG, Leverkusen, Germany, 2.5 mg/kg), the lower back and right knee-joint area were shaved and sterilized with 70% alcohol, and povidone iodine, followed by 250 μL collagenase solution (4 mg/mL collagenase) was then injected intra-articularly in the right knee joint, and this was repeated after 3 days to ensure osteoarthritis. The OA induction method was undertaken as previously described by Huh et al [10]. The rabbits were closely inspected once a day.

### 2.11. Magnetic Resonance Imaging (MRI) Examination

At the end of the experiment (8 weeks) the animals were anesthetized and scanned by Magnetom Essenza 1.5 Tesla (Siemens Co., Munich, Germany) magnetic resonance imaging (MRI). MRI changes were evaluated by the Bouchgua et al. [25] scoring system. Briefly, knee-joint effusion was scored employing grades 0 (normal) to 3 (severe). The severity of osteophytosis was graded from 0 to 3 (normal to severe) at the knee joint. Bone-marrow lesions (BMLs) were graded thus: grade 0, no BMLs; grade 1, lesions involving <25% of the volume of the compartment; grade 2, lesions involving 25–50%; and grade 3, lesions involving >50%. The MRI images were evaluated in a blind manner by a pathologist, who was kept unaware about the treatment groups.

### 2.12. Sample Collection

After anesthesia (as described before in 2.10), at 8 weeks, 8 mL blood was collected from the jugular vein of the subjects into a vacutainer tube containing a clot activator, centrifuged for 10 min at 3000 rpm, solidified at room temperature, then allowed to stand for about 15 min before the sera were separated. Synovial fluid was collected by a 20 gauge syringe from the right knee joint. Serum and synovial fluid were stored in an ultra-low temperature freezer maintained at below about −70 °C until the test was conducted. Under the anesthesia, animals were sacrificed by bleeding from caudal vena cava, and the cartilage samples were harvested and fixed in a 10% neutral buffered formalin solution for histopathological examination.

### 2.13. Histopathological Examination

The fixed tissue was decalcified, embedded in paraffin, and sectioned (5 μm). The sections were stained with hematoxylin-eosin (H-E) and safranin O, then examined under an optical microscope (Olympus BX53, Tokyo, Japan). Histopathological changes were evaluated by a modified technique of the Colombo et al. [26] scoring system. Briefly, for the loss of superficial layer: none 0, minimal 1, mild 2, moderate 3, and marked 4. For ulcer or erosion: none 0, minimal 1, mild 2, moderate 3, and marked 4. For fissure: none 0, minimal 1, mild 2, moderate 3, and marked 4. For loss of proteoglycan: none 0, minimal 1, mild 2, moderate 3, and marked 4. For synovial tissue hyperplasia: none 0, minimal 1, mild 2, moderate 3, and marked 4.

### 2.14. Serum Substance-P and Cytokine Measurement

Substance-P (SP) (Substance-P ELISA Kit, ab133029, Abcam Inc., Cambridge, MA, USA) was measured using serum, and synovial fluid was measured by using IL-1β (Rat IL-1 beta ELISA Kit, Abcam Inc., Cambridge, MA, USA) and TNF-α (Rat TNF alpha ELISA Kit, ab100785, Abcam Inc., Cambridge, MA, USA) according to the company’s protocol.

### 2.15. Statistical Analysis

Data were expressed as means ± standard deviation of the mean (SD). Differences between groups were evaluated by Bonferroni post hoc test following one-way ANOVA. We analyzed the differences by using Prism 5.03 (GraphPad Software Inc., San Diego, CA, USA). Statistical significance was set at *p* < 0.05. The statistical analysis was performed by biostatistician Myung-Jin Kim.

## 3. Results

### 3.1. Cell Viability

**Figure 1:**
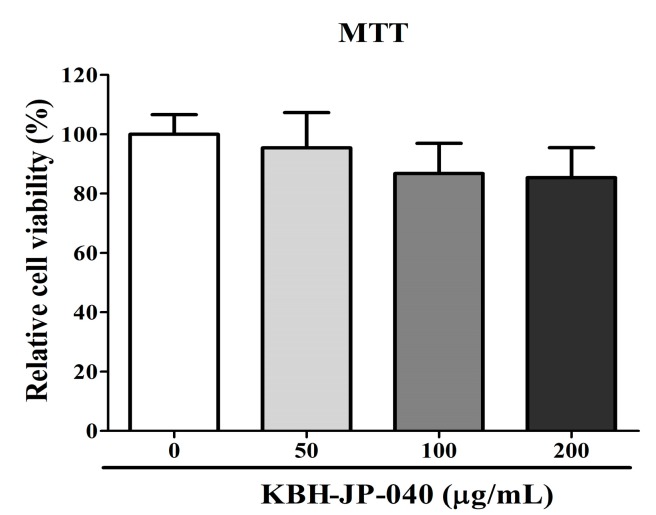
Viability or cytotoxicity test of rat chondrocytes treated with various concentrations of KBH-JP-040 (0, 50, 100, and 200 μg/mL) for 24 h and analyzed by MTT assay and statistically analyzed by Bonferroni post hoc test following one-way ANOVA versus the normal control (NC) group. No significant changes were observed. All experiments were performed using three replicates.

The cytotoxicity of KBH-JP-040 on chondrocytes was judged by MTT assay. All experiments were conducted in triplicate. As shown in Figure 1, KBH-JP-040 at concentration ranging from 10–200 μg/mL did not have significant cytotoxic effects on chondrocytes at the 24 h time point.

### 3.2. Effects of KBH-JP-040 on Aggrecan and Collagen Type-II, PGE2, and ROS

The protein levels of aggrecan and collagen type-II were significantly reduced, while PGE2 and ROS were significantly elevated in rhIL-1α challenged chondrocytes compared to untreated chondrocytes. However, aggrecan and collagen type-II were significantly improved, and PGE2 and ROS were remarkably lowered by KBH-JP-040 treatment when compared with rhIL-1α-challenged chondrocytes, indicating its therapeutic effectiveness (Table 2). 

### 3.3. Effects of KBH-JP-040 on Matrix Metalloproteinase (MMP) and Interleukin (IL) Protein Levels

**Figure 2:**
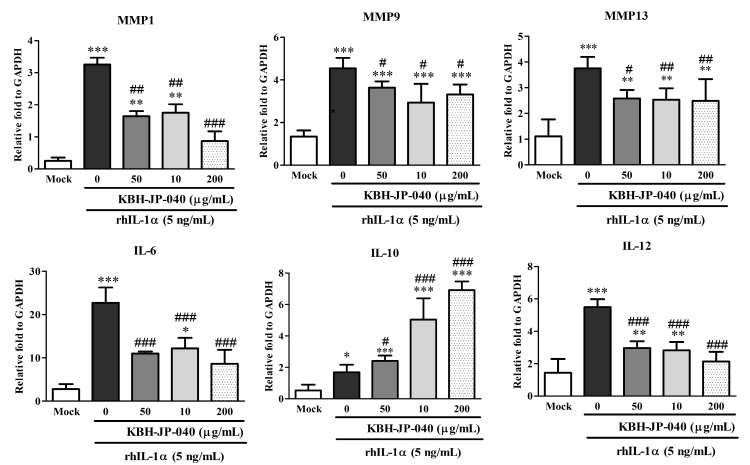
Effect of treatment of KBH-JP-040 on MMP and IL protein expression in rat chondrocytes measured by RT-PCR. Mock, normal chondrocytes; RhIL-1, 5 ng/mL recombinant human IL-1-treated group; RhIL-1+SE50, rhIL-1 and standardized extract , KBH-JP-040 50 μg/mL-treated group; RhIL-1+ KBH-JP-04050, rhIL-1- and KBH-JP-040 50 μg/mL-treated group; RhIL-1+ KBH-JP-040100, rhIL-1- and KBH-JP-040 100 μg/mL-treated group; RhIL-1+ KBH-JP-04050, rhIL-1 and KBH-JP-040 150 μg/mL-treated group. The data are reported as means ± SD. *: *p* < 0.05; **: *p* < 0.01; and ***: *p* < 0.001, Bonferroni post hoc test following one-way ANOVA versus the mock group; #: *p* < 0.05; ##: *p* < 0.01; and ###: *p* < 0.001, Bonferroni post hoc test following one-way ANOVA versus RhIL-1 group. All experiments were performed using three replicates.

The protein levels of MMP-1, MMP-9, MMP-13, IL-6, and IL-12 were significantly (*p* < 0.001) elevated, while that of IL-10 was remarkably lowered in rhIL-1α-challenged chondrocytes compared to untreated chondrocytes. However, MMP-1, MMP-9, MMP-13, IL-6, and 1L-12 were significantly lowered, and IL-10 was remarkably improved by KBH-JP-040 treatment in a dose-dependent manner when compared with rhIL-1α challenged chondrocytes, indicating its therapeutic effectiveness (Table 2, Figure 2).

### 3.4. Effects of KBH-JP-040 on NF-κB, p38, JNK Activation, and IκBα Degradation

**Figure 3:**
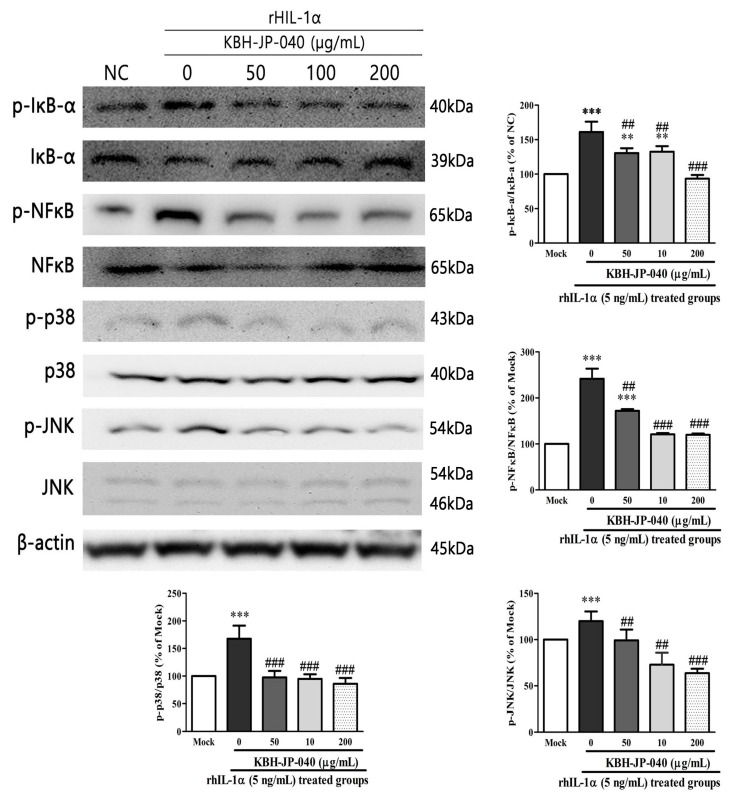
Effect of KBH-JP-040 on pJNK and pp38 MAPK proteins, pIkBα, pNFkB in rhIL-1α-stimulated chondrocytes. The protein expression of pJNK and pp38MAP kinase IkBα, NFkB was analyzed by Western blot analysis. β-Actin was used as an internal control. Mock, normal chondrocytes; RhIL-1, 5 ng/mL recombinant human IL-1-treated group; RhIL-1+ KBH-JP-04050, rhIL-1 and standardized extract, KBH-JP-040 50 μg/mL-treated group; RhIL-1+ KBH-JP-04050, rhIL-1 and KBH-JP-040 50 μg/mL-treated group; RhIL-1+ KBH-JP-040100, rhIL-1- and KBH-JP-040 100 μg/mL-treated group; RhIL-1+ KBH-JP-04050, rhIL-1 and KBH-JP-040 150 μg/mL-treated group. The data are reported as means ± SD. **: *p* < 0.01; and ***: *p* < 0.001, Bonferroni post hoc test following one-way ANOVA versus the mock group; ##: *p* < 0.01; and ###: *p* < 0.001, Bonferroni post hoc test following one-way ANOVA versus RhIL-1 group. All experiments were performed using three replicates.

We next investigated the possible relationship between NF-κB and KBH-JP-040 by determining the related signaling proteins of NF-κB, p38, JNK, and IκBα in rhIL-1α-stimulated chondrocytes. The expression levels of NF-κB, p38, JNK, and IκBα were tested by Western blotting. As shown in Figure 3, KBH-JP-040 notably suppressed the activation of NF-κB, p38, JNK, and the degradation of IκBα induced by rhIL-1α-stimulated chondrocytes.

### 3.5. MRI Analysis

**Figure 4:**
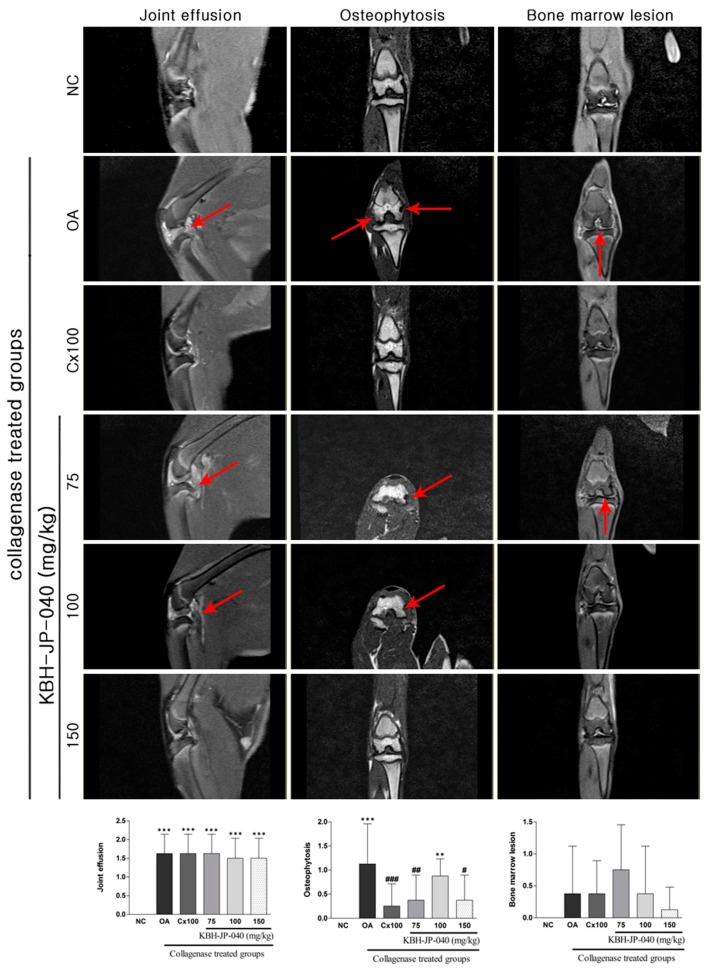
Evaluation of therapeutic efficacy of KBH-JP-040 by MRI images. NC, vehicle-treated normal control group; OA, vehicle-treated collagenase-induced osteoarthritis group; Cx100, collagenase-induced OA rabbits treated with celecoxib 100 mg/kg; collagenase-induced OA rabbits treated with standardized extract, KBH-JP-040-treated groups (75, 100, and 150 mg/kg body weight). Joint effusion, osteophytosis, and bone marrow lesions were (marked by arrow) present in OA group. These alterations were improved in the KBH-JP-040 150 mg/kg- and celecoxib-treated group. The data are reported as means ± SD (*n* = 10). **: *p* < 0.01; and ***: *p* < 0.001, Bonferroni post hoc test following one-way ANOVA versus the NC group; #: *p* < 0.05; ##: *p* < 0.01; and ###: *p* < 0.001, Bonferroni post hoc test following one-way ANOVA versus OA group.

The MRI images of the joints of the KBH-JP-040- and celecoxib (Cx)-treated groups were compared with the knees of the NC and OA groups for BMLs, joint effusion, and osteophyte formation. The osteophytosis scoring system were increased significantly: 1.13±0.30 (*p* < 0.001) in OA group when compared with NC (0) group. However, this pathological alteration was significantly reduced in the Cx (0.25 ± 0.16, −77.88%) and KBH-JP-040, 150 mg/kg (0.38 ± 0.18, −66.81%) when compared with OA group. Joint effusion increased significantly, but was mildly reduced in all treated groups. However, it was most reduced in the KBH-JP-040 150 mg/kg-treated group. BMLs increased, but not significantly. The greatest reduction of BMLs was also found in the KBH-JP-040 150 mg/kg-treated group, indicating its therapeutic efficacy (Figure 4).

### 3.6. Inflammatory Cytokines and Serum Substance-P Level

**Figure 5:**
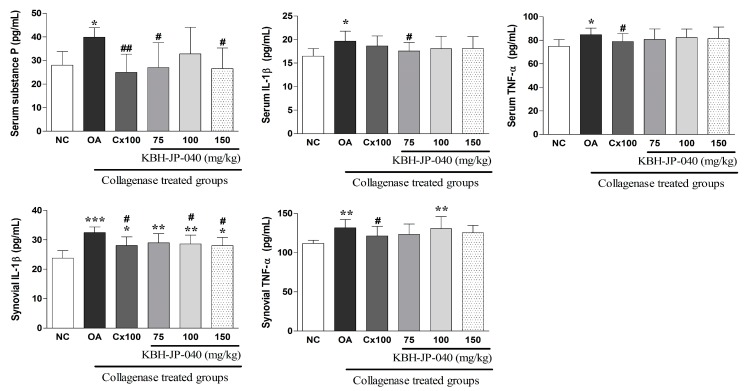
Effect of KBH-JP-040 supplementation on serum substance-P, serum and synovial cytokines of rabbits. NC, vehicle-treated normal control group; OA, vehicle-treated collagenase-induced osteoarthritis group; Cx100, collagenase-induced OA rabbits treated with celecoxib 100 mg/kg; collagenase-induced OA rabbits treated with standardized extract, KBH-JP-040-treated groups (75, 100 and 150 mg/kg body weight). The data are reported as means ± SD (*n* = 10). *: *p* < 0.05; **: *p* < 0.01; and ***: *p* < 0.001, Bonferroni post hoc test following one-way ANOVA versus the NC group; #: *p* < 0.05; ##: *p* < 0.01, Bonferroni post hoc test following one-way ANOVA versus OA group.

Both serum and synovial fluid levels of inflammatory cytokines (IL-6, IL-β) and the serum substance-P level were markedly elevated in OA-induced rats. However, these alterations were successfully corrected in the KBH-JP-040-treated groups (Figure 5).

### 3.7. Histopathological Examination

**Figure 6:**
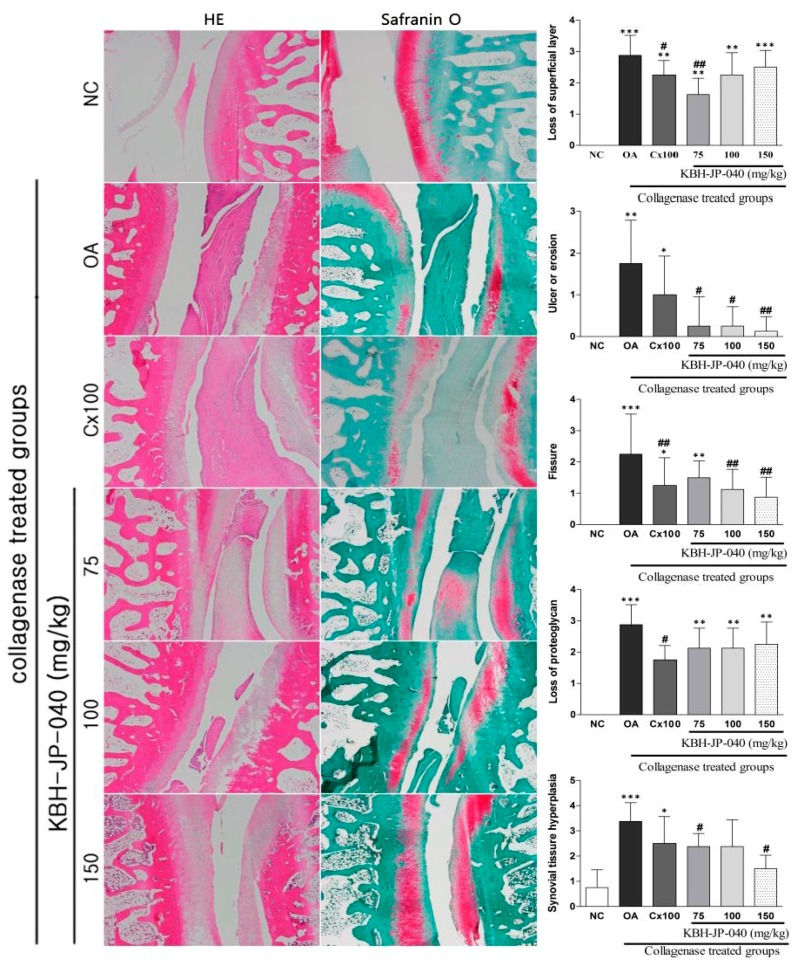
Evaluation of therapeutic efficacy of KBH-JP-040 by histological images of joints. NC, vehicle-treated normal control group; OA, vehicle-treated collagenase-induced osteoarthritis group; Cx100, collagenase-induced OA rabbits treated with celecoxib 100 mg/kg; collagenase-induced OA rabbits treated with standardized extract, KBH-JP-040-treated groups (75, 100, and 150 mg/kg body weight). The data are reported as means ± SD (*n* = 10). *: *p* < 0.05; **: *p* < 0.01; and ***: *p* < 0.001, Bonferroni post hoc test following one-way ANOVA versus the NC group; #: *p* < 0.05; ##: *p* < 0.01, Bonferroni post hoc test following one-way ANOVA versus OA group.

Histopathological examination of hematoxylin and eosin stained results revealed, in the OA group, that lining cells increased in the layer and mononuclear infiltration in the stroma, and the cartilage showed an irregular surface with fibrillation and clefting (Figure 6), degeneration of chondrocytes, and evidence of cloning and focal hypercellularity (Figure 6) when compared with the normal control. Histopathological examination (safranin O stained) showed that the loss of superficial layer, fissure, ulcer or erosion, the loss of proteoglycan and synovial-tissue hyperplasia were all significantly higher in the OA group as compared to the control group. These pathological changes had been ameliorated by the KBH-JP-040 and celecoxib treatment (Figure 6). Moreover, by evaluation of most of the parameters, such as ulcer or erosion, fissure and synovial hyperplasia formation, KBH-JP-040 (150 mg/kg) displayed better chondroprotective effects than celecoxib (Figure 6).

## 4. Discussion

Osteoarthritis is related to a series of inflammatory and pro-inflammatory pathways. In OA, inflammation is accompanied by the destruction of the connective tissue, cartilage, and bone of the joint. Hence, arthritis treatment is targeted at the inflammatory components, leading to the alleviation of pain and swelling [24]. Several studies have demonstrated the anti-inflammatory properties of KP, HE, and AM [15,18,27] and/or their ingredients liriodendrin [16,28], betulin [29,30], and formononetrin [31,32]. Therefore, KBH-JP-040 from HE, KP, and AM was used in experimental rat chondrocytes and rabbit OA models in an effort to reduce inflammation, protect cartilage damage, relieve pain and improve joint pathology. In the vitro study, KBH-JP-040 demonstrated an ability to suppress IL6 and IL12 expression in rhIL-1α-induced rat chondrocytes at both mRNA and protein levels. In addition, the in vivo study also demonstrated that KBH-JP-040 reduced IL-1β and TNF-α both in serum and synovial fluid. This might be due to the anti-inflammatory effects of KBH-JP-040. Moreover, IL10 was elevated markedly by KBH-JP-040 treatment in chondrocytes. Cytokine IL-10 has a chondroprotective effect and stimulates the synthesis of type-II collagen and aggrecan in the course of OA [33]. Collagen II and aggrecan proteins in rat chondrocytes also increased through KBH-JP-040 treatment in this study.

Inflammatory or pro-inflammatory cytokines stimulate chondrocytes to produce MMPs, aggrecanases, and prostaglandins in osteoarthritis [23]. The in vitro study also showed that KBH-JP-040 suppressed MMP-1, MMP-9, and MMP-13 expression in rhIL-1α-induced rat chondrocytes at both mRNA and protein levels. These results indicate that the chondroprotective effects of KBH-JP-040 may be related to the regulation of MMPs. MMPs play critical roles in cartilage degradation due to their ability to cleave a wide variety of components of the extracellular matrix, macromolecules such as aggrecans, fibronectin, and MMP-13, in particular, exhibit activity against collagen type-II, which is the main component of the extracellular matrix [23,34]. Both aggrecans and collagen type-II were improved in KBH-JP-040-treated chondrocytes in this study, which might be due to it regulating inflammation and the MMPs. PGE2, which is an important prostaglandin in osteoarthritis, is relevant to inflammation and its mediators. It is responsible for the classic signs of inflammation—redness, swelling, and pain—through its action of arterial dilatation, microvascular permeability, and sensory-nerve stimulation [35]. It stimulates the production of substance-P via the Ca^2+^-dependent mechanism which is associated with inflammation. In previous studies, PGE2 reduction was identified through treatment with AM [36], HE [37], and KP [38], as well as by liriodendrin [16]. As expected, PGE2 and substance-P were lowered in this case by KBH-JP-040 treatment in the OA model.

In mitogen-activated protein (MAP) kinase process, such as JNK, P38 plays a crucial role in producing MMPs and aggrecanase during OA [39]. We also found that KBH-JP-040 treatment downregulated JNK and p38 MAP kinase phosphorylation in chondrocytes. Thus, KBH-JP-040 might suppress MMP production, and improved aggrecan and collagen type-II consequently prevent cartilage destruction. The NF-κB and I-κB signaling pathways are other important factors in the chondrocyte that regulate the inflammatory response and MMP production, which lead to extracellular matrix damage and cartilage degradation in OA [40]. Therefore, we investigated the effects of KBH-JP-040 on IκBα degradation in order to examine its potential influence on NF-κB. The results showed that KBH-JP-040 could also downregulate NF-κB and I-κB protein expression in rhIL-1α-induced chondrocytes, which also mirrored the chondroprotective mechanism of KBH-JP-040.

It is worth pointing out that the pharmacological functions of many compounds from KP, especially kalopanaxsaponin-A, have been shown to inhibit the overexpression of TNF-α and IL-1β and downregulation of COX-2 expression via modulating NF-κB/AP-1 [17,18,41]; and that HE treatment suppressed TNF-α-induced overexpression of MMP-9 and downregulated the nuclear translocation and transcriptional activation of NF-κB, followed by suppression of I-κB degradation [15]. Friedman [21] also reported that HE extracts reduce the expression of the matrix metalloproteinases MMP-2, MMP-9, COX-2, and 5-LOX in cells, which are all overexpressed in arthritic conditions. Treatment with these herbals or their bioactive ingredients reduced LPS-induced pro-inflammatory mediators, such as IL-1β, IL-6, and TNF-α and oxidative stress [30,31,36]. The bioactive component of AM was identified as possessing an anti-inflammatory property by reducing the release of inflammatory mediators and deactivating NF-κB through the MAPK signaling pathway [36]. Therefore, it is noteworthy that KBH-JP-040 has the qualities of ameliorating biochemical changes and exerting chondroprotective effects, which were confirmed in this study. 

ROS were suppressed in the treatment group in this study. Several studies have attributed anti-oxidant activities to KP [42], HE [21] and AM [27,43]. Through this property, the combined extract might also protect against tissue injuries in the joint by regulating free radicals/ROS in cells, as reflected by the improved histopathology and MRI results in the treatment group. Better chondroprotective effects were found by histopathological examination, such as less ulcers or erosion, and fissure and synovial hyperplasia formation in KBH-JP-040 (150 mg/kg)-treated group compared to celecoxib treatment, possibly due to its active bio-ingredients. Joint effusion, osteophytosis, and BMLs are important parameters to detect pathological alterations of arthritis [25,44,45]. To the best of our knowledge, this is the first time that joint effusion, osteophytosis, and BMLs have been reported in collagenase-induced rabbit OA model. The most reduction of joint effusion and BMLs were also observed in the KBH-JP-040 150 mg/kg-treated group, indicating its therapeutic superiority. These findings also support the molecular results obtained in this study, and further confirm the chondroprotective effects of KBH-JP-040.

## 5. Conclusions

In conclusion, in vitro results show that KBH-JP-040 protects cartilage by suppressing inflammation and MMPs and by downregulating IκBα, NF-κB, JNK/p38 MAP kinase signaling pathways. In vivo experiments also confirmed its therapeutic efficacy through improved biochemical, MRI, and histopathological results, which were similar or more effective than the celecoxib-treated group. Therefore, the results indicate that this herbal mixture possesses chondroprotective effects, and may be a potential safe herbal therapeutic agent for the treatment of OA.

## Figures and Tables

**Table 1:** Primer sequences utilized for real time RT-PCR evaluation of gene expression.

	Forward	Reverse
Collagen type 2	5′ CCG ATC CCC TGC AGT ACA TG 3′	5′ TGC TCT CGA TCT GGT TGT TCA 3′
SOX9	5′ CTG AAG GGC TAC GAC TGG AC 3′	5′ TAC TGG TCT GCC AGC TTC CT 3′
MMP1	5′-CTC CCT TGG ACT CAC TCA TTC TA-3	5′-AGA ACA TCA CCT CTC CCC TAA AC-3′
MMP2	5′-TGG GGG AGA TTC TCA CTT TG 3′	5′CCA TCA GCG TTC CCA TAC TT 3′
MMP3	5′ TGG GAA GCC AGT GGA AAT G 3′	5′CCA TGC AAT GGG TAG GAT GAG 3′
MMP9	5′ TGC TCC TGG CTC TAG GCT AC 3′	5′ TTG GAG GTT TTC AGG TCT CG 3′
MMP13	5′ CTG ACC TGG GAT TTC CAA AA 3′	5′ ACA CGT GGT TCC CTG AGA AG 3′
IL-6	5′ TGATGGATGCTTCCAAACTG 3′	5′GAGCATTGGAAGTTGGGGTA 3′
IL-10	5′ GAGAGAAGCTGAAGACCCTCTG 3′	5′TCATTCATGGCCTTGTAGACA C 3′
IL-12	5′-AGG CCC AGC AGC AGA ATA AAT A-3′	5′-GTG CTC CAG GAG TCA GGG TAC T-3′
GAPDH	5′ GG GTG TGA ACC ACG AGA AAT 3′	5′ ACT GTG GTC ATG AGC CCT TC 3′

**Table 2:** Effect of standardized extract (KBH-JP-040) on aggrecan, collagen type-II, inflammatory cytokines, matrix metaloproteins, PGE2 and ROS generation in rat chondrocytes.

	NC	RhIL-1	RhIL-1+KBH-JP-040 50	RhIL-1+KBH-JP-040100	RhIL-1+KBH-JP-040200
Aggrecan (ng/mL)	12.88 ± 0.62	10.90 ± 0.60 **	11.54 ± 1.30 *	11.57 ± 1.05 **^,##^	11.74 ± 1.06 **^,#^
Collagen type II (ng/mL)	12.85 ± 1.15	9.51 ± 0.48 **	11.80 ± 0.54 **^,#^	12.93 ± 3.81 *^,##^	13.32 ± 1.22 *^,##^
IL-6 (pg/mL)	35.20 ± 8.24	60.30 ± 0.76	51.66 ± 2.44	51.24 ± 1.60	50.63 ± 5.32
IL-10 (pg/mL)	3.34 ± 2.06	3.53 ± 1.66 ***	4.00 ± 1.43 **	4.79 ± 2.95 *^,#^	4.65 ± 3.35 *
IL-12 (pg/mL)	1.32 ± 0.67	2.30 ± 0.64 **	1.51 ± 0.36 *^,##^	1.39 ± 0.24 ^###^	1.01 ± 0.52 ^###^
MMP1 (pg/mL)	52.98 ± 10.69	93.06 ± 34.37 ***	46.19 ± 30.69 ***^,#^	38.31 ± 15.35 **^,###^	41.37 ± 12.95 **^,###^
MMP9 (pg/mL)	34.82 ± 12.80	54.05 ± 10.05 ***	39.24 ± 4.94 ***^,#^	38.43 ± 5.88 **^,###^	33.21 ± 3.45 **^,###^
MMP13 (pg/mL)	189.35 ± 23.33	269.50 ± 37.08 **	211.45 ± 20.37 *	208.25 ± 38.84 ^#^	192.15 ± 38.73
PGE2 (pg/mL)	58.30 ± 1.33	81.78 ± 1.19 **	54.32 ± 0.81 *	41.20 ± 0.69 ^#^	47.22 ± 2.50
ROS (%)	545.20 ± 17.25	645.40 ± 36.73 ***	563.20 ± 6.45 **^,#^	567.60 ± 11.01 *^,##^	555.60 ± 13.43 **^,#^

NC, normal chondrocytes; RhIL-1, 5 ng/mL recombinant human IL-1-treated group; RhIL-1+ KBH-JP-04050, rhIL-1 and standardized extract , KBH-JP-040 50 μg/mL-treated group; RhIL-1+ KBH-JP-04050, rhIL-1 and KBH-JP-040 50 μg/mL-treated group; RhIL-1+ KBH-JP-040100, rhIL-1 and KBH-JP-040 100 μg/mL-treated group; RhIL-1+ KBH-JP-04050, rhIL-1 and KBH-JP-040 150 μg/mL-treated group. IL interleukin; MMP matrix metalloproteinases, PGE2 prostaglandins E2; ROS reactive oxygen species. All experiments were performed using three replicates. The data are reported as means ± SD. *: *p* < 0.05; **: *p* < 0.01; and ***: *p* < 0.001, Bonferroni post hoc test following one-way ANOVA versus the mock group; ^#^: *p* < 0.05; ^##^: *p* < 0.01; and ^###^: *p* < 0.001, Bonferroni post hoc test following one-way ANOVA versus RhIL-1 group.
